# Efficacy of small molecules against the severe acute respiratory syndrome coronavirus 2 XBB1.16 and XBB1.9.2.1

**DOI:** 10.1002/mco2.500

**Published:** 2024-02-28

**Authors:** Lunzhi Yuan, Xuan Liu, Song Li, Wu Zhong, Ningshao Xia

**Affiliations:** ^1^ State Key Laboratory of Vaccines for Infectious Diseases, National Institute of Diagnostics and Vaccine Development in Infectious Diseases, NMPA Key Laboratory for Research and Evaluation of Infectious Disease Diagnostic Technology, School of Life Sciences & School of Public Health Xiamen University Xiamen China; ^2^ Clinical Center for Bio‐Therapy, Zhongshan Hospital Fudan University (Xiamen Branch) Xiamen China; ^3^ National Engineering Research Center for the Emergency Drug Beijing Institute of Pharmacology and Toxicology Beijing China

Dear Editor,

The Omicron variant (B.1.1.529) of severe acute respiratory syndrome coronavirus 2 (SARS‐CoV‐2) and hundreds of its progenies have rapidly emerged as the predominant circulating viruses worldwide. Recognized as variants of interest and variants under surveillance (VUMs) by the World Health Organization, these variants strategically evade the immune response instigated by natural infection or vaccination.^1^, ^2^, ^3^ This evasion mechanism stems from intensive mutations within the receptor binding domain (RBD) and N‐terminal domain (NTD) of the spike protein, thereby bolstering the ability to circumvent neutralizing antibodies and heightening the incidence of breakthrough infections (BTIs). Conversely, small‐molecule antiviral drugs that inhibit viral replication, such as RNA‐dependent RNA polymerase inhibitors and protease inhibitors like remdesivir, molnupiravir, and nirmatrelvir, consistently maintain robust efficacy against a broad spectrum of variants assessed in cell cultures.^1^, ^2^, ^3^ These antiviral options present supplementary avenues for combating BTIs, facilitating in‐house treatment, and alleviating the severity rate among hospitalized patients. However, the naturally occurring mutations of SARS‐CoV‐2 main protease confer drug resistance to nirmatrelvir,^4^ which suggests the urgent need for evaluation of risk of drug resistant.

Recently, several Omicron sublineages of SARS‐CoV‐2, specifically XBB1.16 and XBB1.9, have emerged prominently. Their subsequent generations, encompassing XBB1.16.1/1.16.6, XBB1.9.1/1.9.2, XBB1.9.2.1 (EG.1), and XBB1.9.2.5.1 (EG.5.1), have collectively contributed to more than 60% of the global SARS‐CoV‐2 infections. In contrast to the earlier prevailing sublineage XBB1.5, XBB.1.16 showcases a dual substitution phenomenon in both nonstructural proteins (NSPs) and the spike protein. Specifically, T478R mutation within the RBD and E180V mutation within the NTD define its genetic makeup. As expected, XBB.1.16 displays notable resistance to various anti‐SARS‐CoV‐2 antibodies. Differently, XBB1.9.2 introduces a pair of substitutions in the NSPs, T35I in NSP9 and N1001S in NSP3, implying that mutations beyond the spike protein might contribute to an elevated efficiency of viral replication.

Notably, Omicron/XBB BTIs exhibit insufficient stimulation of antiviral humoral immunity against XBB variants, thereby underscoring the imperative of alternative strategies to hinder infection by these emerging XBB variants and their successors.^5^, ^6^ Indeed, our data confirmed that SARS‐CoV‐2 Omicron XBB1.16 and XBB1.9.2.1 variants can partially escape neutralization effect of the convalescent serum of hamsters that infected with SARS‐CoV‐2 Omicron BA.1 or BA.5 (Figure S1). Furthermore, significant body weight loss, robust viral replication in respiratory tract organs, and severe lung injury were observed in hamster infected with XBB1.9.2.1 by a route of close contact infection (Figure S1), which indicates increased pathogenicity.

In this study, we evaluated the efficacy of seven small‐molecule antiviral compounds, including GS‐441524 (the primary metabolite of remdesivir), nirmatrelvir (also known as PF‐07321332), VV116 (an oral derivative of remdesivir), EIDD‐1931 (the active form of molnupiravir), EIDD‐2801 (molnupiravir), BAY2402234 (an inhibitor of dihydroorotate dehydrogenase, DHODH), and SYM‐3‐11 (ensitrelvir). We conducted these assessments using cell cultures infected with the prototype SARS‐CoV‐2 and variants include 614G, Beta, Delta, Omicron BA.1/BA.5, XBB1.16, and XBB1.9.2.1. The evaluation was based on the determination of the 50% inhibitory concentration (IC50), and the results showed significant efficacy of all seven small‐molecule antivirals against the tested viruses (Figure S2). GS‐441524, nirmatrelvir, VV116, EIDD‐1931, EIDD‐2801, BAY2402234, and SYM‐3‐11 (ensitrelvir) exhibited IC50 values ranging from 1.008 to 2.850, 1.595 to 4.742, 0.367 to 1.005, 0.146 to 1.368, 0.415 to 2.788, 0.002 to 0.063, and 0.006 to 0.156 μM against both the prototype virus and all the tested variants (Figure 1A).

**FIGURE 1:**
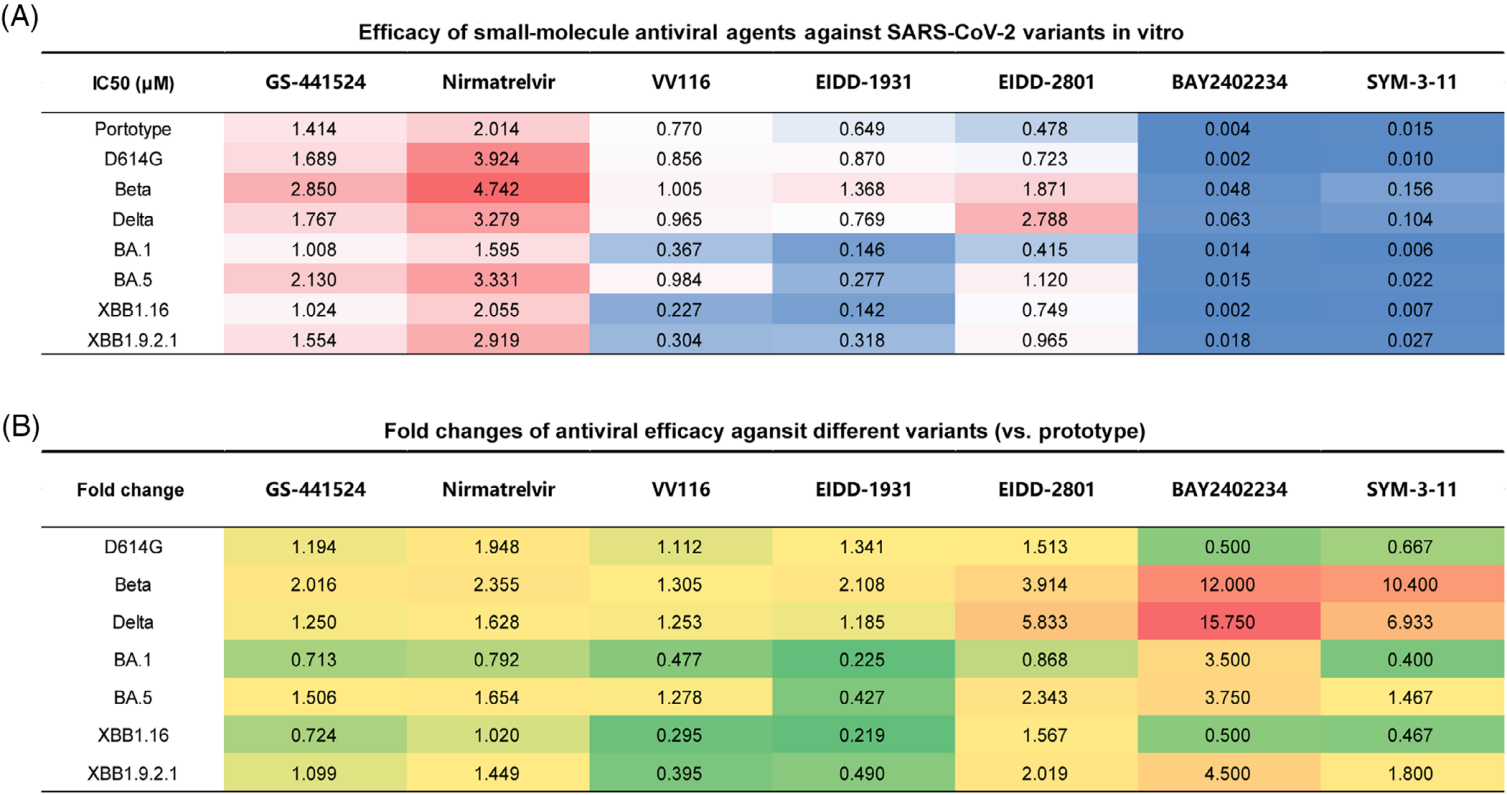
Evaluation for the in vitro antiviral efficiency of small‐molecule antiviral agents against the prototype severe acute respiratory syndrome coronavirus 2 (SARS‐CoV‐2) and variants. (A) The 50% inhibitory concentration (IC50) values of indicated drug–virus groups were calculated and listed accordingly (*n* = 4). Red color indicates a relative lower antiviral efficiency and blue color indicates a relative higher antiviral efficiency. (B) The fold changes of antiviral efficiency against different variants (vs. the prototype) were shown. Red color indicates an increase in IC50 value and a decrease in antiviral efficiency. Green color indicates a decrease in IC50 value and an increase in antiviral efficiency.

When tested against the XBB1.16 variant, these agents displayed IC50 values of 1.024, 2.055, 0.227, 0.142, 0.749, 0.002, and 0.007 μM, and IC50 values of 1.554, 2.919, 0.304, 0.318, 0.965, 0.018, and 0.027 μM against the XBB1.9.2.1 variant (Figure 1A). The fold changes of antiviral efficiency against different variants (vs. the prototype) showed all the antiviral agents provide considerable inhibition against all the tested variants (Figure 1B). Moreover, the recently introduced agents, including BAY2402234 and SYM‐3‐11, demonstrated potent antiviral effectiveness against all tested viruses, exceeding those reported in the initial stages of the SARS‐CoV‐2 pandemic. Different from the other six compounds that directly inhibit the process of viral replication, BAY2402234 is a host‐target compound, effectively inhibiting the activity of a pivotal enzyme (DHODH) in the fourth step of the de novo pyrimidine synthesis pathway. These findings emphasize that both the approaches of direct‐acting and host‐targeting strategies show potential in the development of broad‐spectrum antiviral solutions against the evolving SARS‐CoV‐2. Moreover, the recently introduced agents, including BAY2402234 and SYM‐3‐11, demonstrated potent antiviral effectiveness against all tested viruses, exceeding those reported in the initial stages of the SARS‐CoV‐2 pandemic.

Collectively, a wide array of small molecules, each with unique targets and antiviral mechanisms, play a crucial role in the management and treatment of SARS‐CoV‐2 infections. Recognizing the potential for viral evolution to result in unforeseen changes to its virological characteristics and immune evasion capabilities, there is an urgent need to establish a timely method for evaluating the antiviral efficacy of both approved and recently introduced small‐molecule agents against the prevailing VOCs and VUMs. Considering XBB1.9.2.1 is the parent virus of EG.5/EG.5.1, our data suggest that the tested drugs might keep high antiviral efficiency against these two emerging variants with an increasing proportion in all the reported circulating SARS‐CoV‐2. In addition, long‐term evaluation in respiratory tract organoids and animal models are necessary steps to know whether these drugs can prevent virus mutation in human with SARS‐CoV‐2 infections. Overall, this proactive approach will prove indispensable in anticipating the emergence of drug resistance and guiding clinical interventions.

## AUTHOR CONTRIBUTIONS

L. Y. and X. L. contributed equally to this work. L. Y. and X. L. performed the cell and animal experiments. S. L. and W. Z. provided the small molecules and designed the antiviral studies. L. Y., X. L., and W. Z. wrote the manuscript. N. X. supervised the study. All the authors have read and approved the final manuscript.

## CONFLICT OF INTEREST STATEMENT

The authors declare no conflicts of interest.

## ETHICS STATEMENT

In this study, the virus and animal studies were approved by the ethics committee of the Guangdong‐Hong Kong Joint Laboratory of Emerging Infectious Diseases/Joint Laboratory for International Collaboration in Virology and Emerging Infectious Diseases (Key Laboratory of Ministry of Education), Joint Institute of Virology (Shantou University/The University of Hong Kong) (Approval number: SUMC2022‐051).

## Supporting information

Supporting Information

## Data Availability

The information of supplemental tables, methods and materials were shown in the Supplementary data file. Further information and requests for resources and reagents should be directed to and will be fulfilled by the corresponding author.
