# Supplementary material for: Efficacy of small molecules against the severe acute respiratory syndrome coronavirus 2 XBB1.16 and XBB1.9.2.1
Source: MedComm (2020). 2024 Feb 28;5(3):e500. doi: 10.1002/mco2.500 (PMC10901279; doi:10.1002/mco2.500)
Supplement: Supplementary file 1 — Supporting Information [file MCO2-5-e500-s001.docx]

Supplementary Data

**Efficacy of small molecules against the SARS-CoV-2 XBB1.16 and XBB1.9.2.1**

Lunzhi Yuan^1#^*, Xuan Liu^2#^, Song Li^3^, Wu Zhong^3^*, Ningshao Xia^1^*

^1^State Key Laboratory of Vaccines for Infectious Diseases, National Institute of Diagnostics and Vaccine Development in Infectious Diseases, NMPA Key Laboratory for Research and Evaluation of Infectious Disease Diagnostic Technology, School of Life Sciences & School of Public Health, Xiamen University, Xiamen, Fujian, China

^2^Clinical Center for Bio-Therapy, Zhongshan Hospital, Fudan University (Xiamen Branch), Xiamen, Fujian, China

^3^National Engineering Research Center for the Emergency Drug, Beijing Institute of Pharmacology and Toxicology, Beijing, China

**Supplementary Figure and Figure Legend**


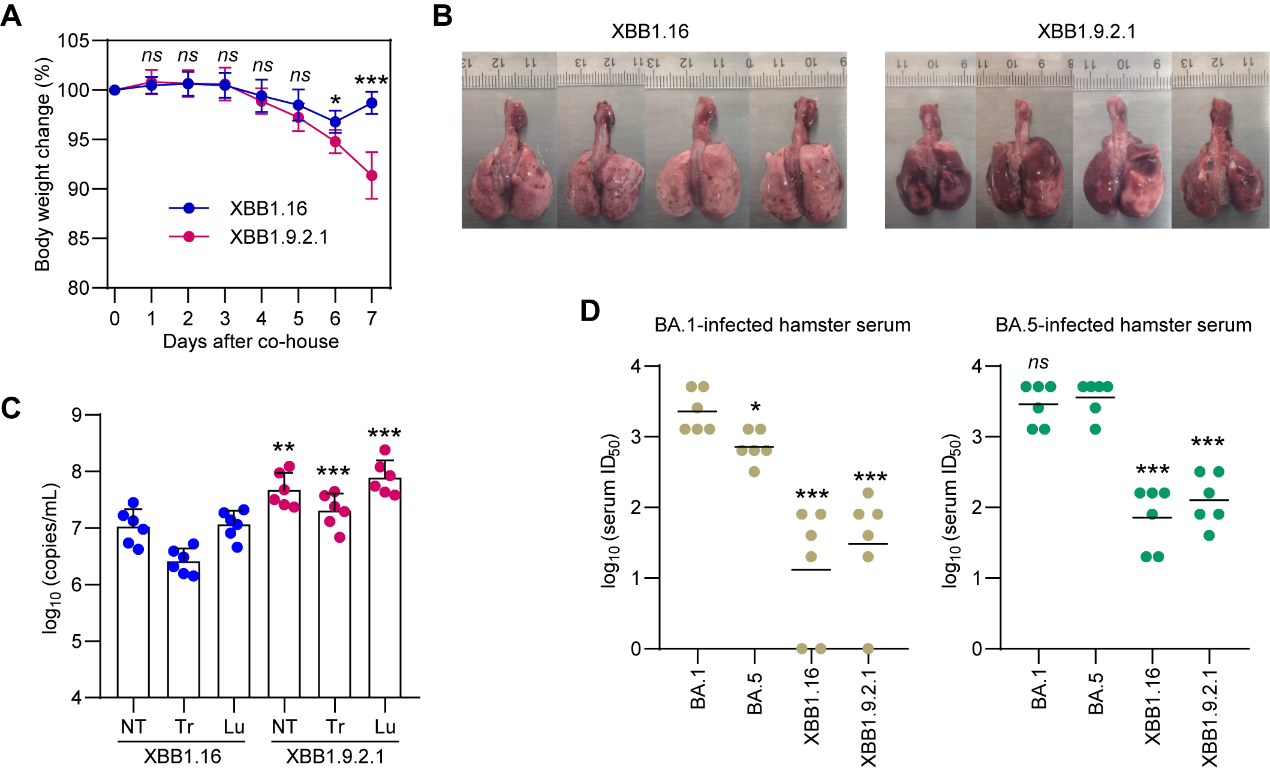


**Figure 1. Virological characteristics of SARS-CoV-2 Omicron XBB1.16 and XBB1.9.2.1 variants in hamster model. (A)** Body weight changes of the hamsters infected with SARS-CoV-2 Omicron XBB1.16 and XBB1.9.2.1 variants by the route of co-house transmission (n=6/group). **(B)** Representative gross images of the lung tissues collected from hamsters that euthanized at 7 days post infection (n=4/group). **(C)** Viral RNA levels from turbinate (NT), trachea (Tr) and lung (Lu) tissues were measured by RT-PCR (n=6/group), using primers to amplify the SARS-CoV-2 ORF1ab gene. **(D)** The variant-specific neutralization titers against SARS-CoV-2 Omicron BA.1, BA.5, XBB1.16 and XBB1.9.2.1 variants were measured by a titration method based on a titration method of TCID50 inhibition. Serum samples collected from hamsters that infected with SARS-CoV-2 Omicron BA.1 (left) or BA.5 (right) were tested(n=6/group). Statistical analysis of body weight data was calculated by two-way ANOVA. Statistical analysis of viral load and serum Neutralization titer was calculated by one-way ANOVA. Two-sided p values <0.01 were considered significant: *P <0.01, **P <0.001, ***P <0.0001, ns indicates no significance.


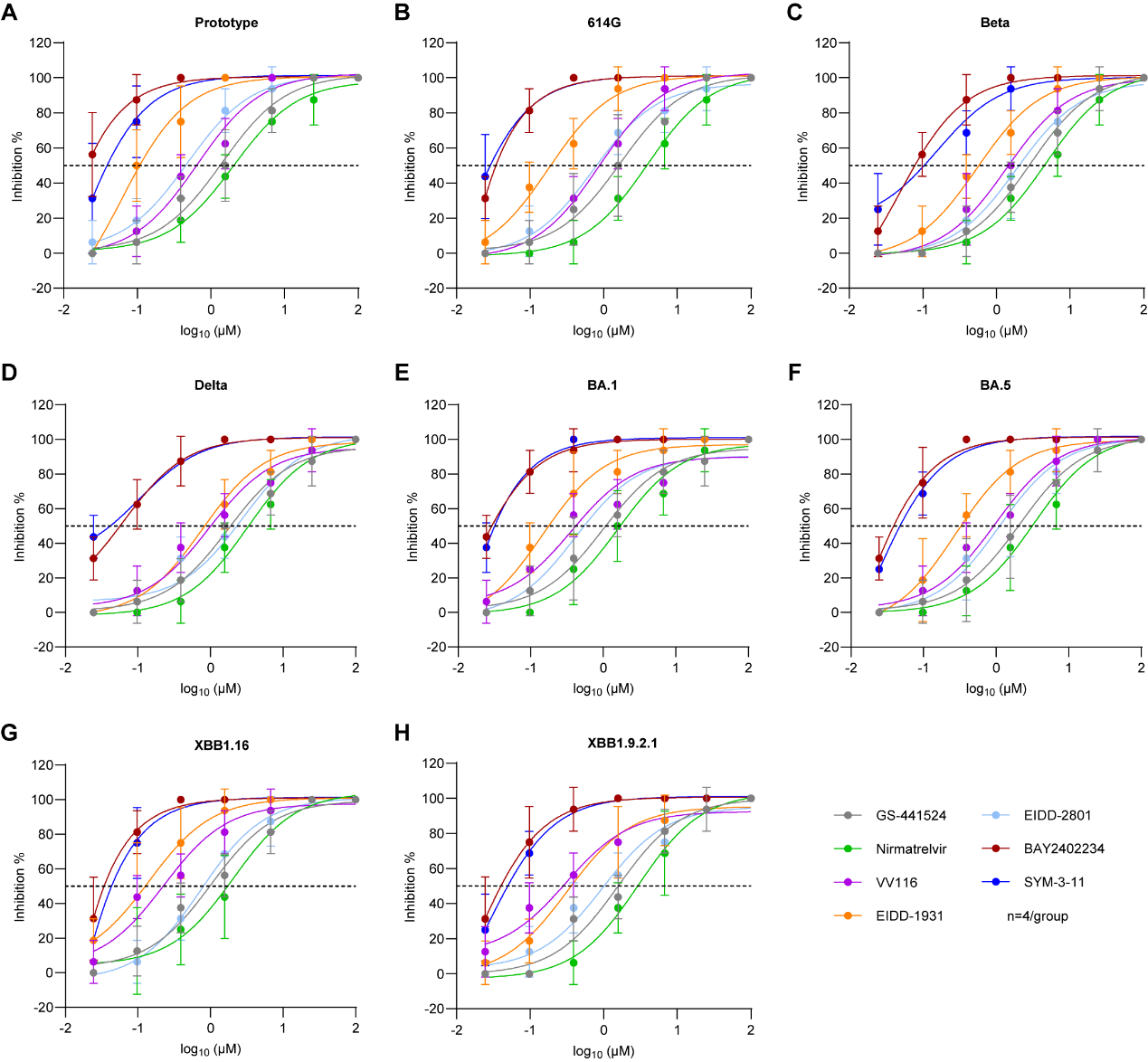


**Figure S2. Evaluation for the in vitro antiviral efficiency of small molecule antiviral agents against the prototype SARS-CoV-2 and variants.** The antiviral efficiency of the seven tested agents including GS-441524, Nirmatrelvir, VV116, EIDD-1931, EIDD-2801, BAY2402234 and SYM-3-11 were tested in 96-well plates seeded with Vero cells. The relationship curves of diluted drug concertation and percent inhibition of cytopathic effect in the **(A)** prototype virus and variants include **(B)** 614G, **(C)** Beta, **(D)** Delta, **(E)** Omicron BA.1, **(F)** BA.5, **(G)** XBB1.16 and **(H)** XBB1.9.2.1 were presented, respectively. For each test, four-well repeat was performed.

**Supplementary Materials and Methods**

**Viruses and biosafety**

The prototype SARS-CoV-2 (AP-8, EPI_ISL_1655937) and variants include 614G (AP-62, EPI_ISL_2779639), Beta (AP-100, EPI_ISL_2779638), Delta (AP-1914, EPI_ISL_2385091), Omicron BA.1 (AP-309, EPI_ISL_8182026), BA.5 (AP-101, share an identical sequence with EPI_ISL_12920651), XBB1.16 (AP-143, share an identical sequence with EPI_ISL_17684413) and XBB1.9.2.1 (AP-144, share an identical sequence with EPI_ISL_17660518) were used in this study. All the viruses were propagated in Vero cells (#CCL-81, ATCC). The virus titers were determined by means of plaque formation unit (PFU) and 50% tissue culture infective dose (TCID50) in Vero cells.

All experiments with infectious SARS-CoV-2 were performed in the biosafety level 3 (BSL-3) facilities. Our staff wore powered air-purifying respirators that filtered the air, and disposable coveralls when they cultured the virus and handled animals that were in isolators. The researchers were disinfected before they left the room and then showered on exiting the facility. All facilities, procedures, training records, safety drills, and inventory records were subject to periodic inspections and ongoing oversight by the institutional biosafety officers who consult frequently with the facility managers. All the animal experiments were approved by the Medical and Animal Ethics Committee (SUMC2022-051).

**Cell cultures**

Vero cells were cultured with Dulbecco's modified Eagle's medium (DMEM, #11995, GBICO) containing 10% fetal bovine serum (FBS, #10270106, GBICO), 100 U/mL Penicillin-Streptomycin (Invitrogen, #15140-122) and 30 mmol/L MgCl_2_ (Thermo-Fisher, #AM9530G), and were maintained at 37 °C with 5% CO_2_. FBS-free DMEM with 5ug/mL TPCK-trypsin (SIGMA-ALDRICH, #T1426) was used for virus propagation.

**Detection of virus titer**

The amount of infective viral particles in the tested sample indicates viral titer, which are usually measured by half tissue culture infective dose (TCID50) titration method in Vero cells seeded in 96-well plates. In the TCID50 titration assay, Vero cells were incubated with 100μL of samples and 10-fold serial diluted samples for one hour. And then, we renewed fresh medium and observed cytopathic effect (CPE) at three days after incubation. Generally, phenomena such as cell rounding, necrosis, and detachment from the bottle wall of plate are representative features of CPE. We defined that all cells without cytopathic effect indicate “zero”.

**Small molecule antiviral agents**

The tested small molecule antiviral agents include GS-441524 (MCE，#HY-103586), Nirmatrelvir (AOSAIKANG, #220202), VV116 (Shanghai Junshi, 220301C051), EIDD-1931 (Shanghai Biochempartner, #20210809), EIDD-2801 (Shanghai Biochempartner, #20210302), BAY2402234 (MCE, #HY-112645) and SYM-3-11 (Ensitrelvir, National Engineering Research Center for the Emergence Drugs, #010121) were dissolved in dimethyl sulfoxide (DMSO, SIGMA-ALDRICH, #D5879) at the concentration of 1 mM for further dilution.

**Inhibitory effect of agents against SARS-CoV-2 in vitro.**

The antiviral susceptibilities of the tested agents were determined by inhibition of cytopathic effect. The Vero cells seeded in 96-well plates were inoculated with 100 TCID50/well of indicated virus for one hours at 37 °C, and then the inoculum was removed. FBS-free DMEM containing serial dilutions of indicated agent were added in the wells. Three days after that, observation of cell cytopathic effect was performed. The results are expressed as the 50% inhibitory concentration (IC50). The IC50 values

were calculated by using GraphPad Prism (GraphPad Software).

**Virus inoculation, co-house transmission and sample collection**

Six- to eight-week-old male hamsters were anesthetized by isoflurane (RWD Life Science, #R510-22) and nasally inoculated with indicated doses of SARS-CoV-2 diluted in 200μL of PBS (GIBCO, #10010031). The hamster inoculated with 1×10^4^ PFU of SARS-CoV-2 was set as indexer. After that, six naïve hamsters were co-housed with two indexers in an isocage. Body weight of the co-housed naïve hamsters were measured by an electronic balance. The co-housed naïve hamsters were euthanized at the seven days post infection for collection of serum and detection of viral load in respiratory tract organs. In this study, we collected 1 gram of turbinate, 0.1 gram of trachea and lung tissues for detection of viral RNA.

**Detection of viral RNA in tissue samples and serum neutralization titers**

Viral RNA was extracted by using a QIAamp Viral RNA Mini kit (#52906, Qiagen) according to the manufacturer's instructions. The RT-PCR was conducted by using the SLAN-96S Real-Time System (Hongshi, Shanghai, China) with a SARS-CoV-2 RT-PCR Kit from Wantai (WS-1248, Beijing, China). Relative Viral RNA of SARS-CoV-2 ORF1ab gene was determined using primer pairs and probes provided in the kit. Viral RNA copies were expressed on a log_10_ scale after normalized to the standard curve obtained by using ten-fold dilutions of a SARS-CoV-2 stock. The serum neutralization titers were measured by a titration method based on TCID50 inhibition. We added 10 μL of serum sample in 90 μL of medium for each well and performed 2-fold gradient dilution. After that, 100 μL of serum sample and 100 TCID50 virus in 100 μL medium were co-incubated with Vero cells for one hour. Finally, we renewed fresh medium and observed inhibition of CPE at three days after co-incubation.
